# Hadaka Virus 1: a Capsidless Eleven-Segmented Positive-Sense Single-Stranded RNA Virus from a Phytopathogenic Fungus, Fusarium oxysporum

**DOI:** 10.1128/mBio.00450-20

**Published:** 2020-05-26

**Authors:** Yukiyo Sato, Wajeeha Shamsi, Atif Jamal, Muhammad Faraz Bhatti, Hideki Kondo, Nobuhiro Suzuki

**Affiliations:** a Institute of Plant Science and Resources, Okayama University, Kurashiki, Japan; b Atta-ur-Rahman School of Applied Biosciences (ASAB), National University of Sciences and Technology (NUST), Sector H-12, Islamabad, Pakistan; c Crop Diseases Research Institute, National Agricultural Research Centre, Islamabad, Pakistan; National Institutes of Health

**Keywords:** fungal virus, polymycovirus, *Fusarium oxysporum*, multisegmented, RNA virus, capsidless, neo-virus lifestyle

## Abstract

The search for viruses infecting fungi, or mycoviruses, has extended our knowledge about the diversity of RNA viruses, as exemplified by the discovery of polymycoviruses, a phylogenetic group of multisegmented RNA viruses with unusual forms. The genomic RNAs of known polymycoviruses, which show a phylogenetic affinity for animal positive-sense single-stranded RNA [(+)RNA] viruses such as caliciviruses, are comprised of four conserved segments with an additional zero to four segments. The double-stranded form of polymycovirus genomic RNA is assumed to be associated with a virally encoded protein (proline-alanine-serine-rich protein [PASrp]) in either of two manners: a capsidless colloidal form or a filamentous encapsidated form. Detailed molecular characterizations of polymycoviruses, however, have been conducted for only a few strains. Here, a novel polymyco-related virus named Hadaka virus 1 (HadV1), from the phytopathogenic fungus Fusarium oxysporum, was characterized. The genomic RNA of HadV1 consisted of an 11-segmented positive-sense RNA with highly conserved terminal nucleotide sequences. HadV1 shared the three conserved segments with known polymycoviruses but lacked the PASrp-encoding segment. Unlike the known polymycoviruses and encapsidated viruses, HadV1 was not pelleted by conventional ultracentrifugation, possibly due to the lack of PASrp. This result implied that HadV1 exists only as a soluble form with naked RNA. Nevertheless, the 11 genomic segments of HadV1 have been stably maintained through host subculturing and conidiation. Taken together, the results of this study revealed a virus with a potential novel virus lifestyle, carrying many genomic segments without typical capsids or PASrp-associated forms.

## Abstract (precis)

Fungi collectively host various RNA viruses. Examples include encapsidated double-stranded RNA (dsRNA) viruses with diverse numbers of genomic segments (from 1 to 12) and capsidless viruses with nonsegmented (+)RNA genomes. Recently, viruses with unusual intermediate features of an infectious entity between encapsidated dsRNA viruses and capsidless (+)RNA viruses were found. They are called polymycoviruses, which typically have four to eight dsRNA genomic segments associated with one of the virus-encoded proteins and are phylogenetically distantly related to animal (+)RNA caliciviruses. Here, we identified a novel virus phylogenetically related to polymycoviruses, from the phytopathogenic fungus Fusarium oxysporum. The virus, termed Hadaka virus 1 (HadV1), has 11 (+)RNA genomic segments, the largest number in known (+)RNA viruses. Nevertheless, HadV1 lacked a typical structural protein of polymycoviruses and was not pelleted by standard ultracentrifugation, implying an unusual capsidless nature of HadV1. This study reveals a potential novel lifestyle of multisegmented RNA viruses.

## INTRODUCTION

In 1970, Baltimore classified RNA viruses, utilizing RNA-dependent RNA polymerases (RdRPs) for their replication, into three groups based on their genome types: double-stranded RNA (dsRNA), positive-sense single-stranded RNA [(+)RNA], and negative-sense single-stranded RNA [(−)RNA] (1). Recent virus metagenomics and conventional virus hunting have greatly extended our knowledge about the diversity and evolution of these RNA viruses (2, 3). Wolf et al. suggested that RNA viruses can be grouped into five branches, I to V, based on improved phylogenetic relations with RdRP sequences (4), one (branch II, picornavirus-like supergroup) of which accommodates both dsRNA and (+)RNA viruses. This has prompted the International Committee on Taxonomy of Viruses (ICTV) (https://talk.ictvonline.org) to create the realm *Riboviria*, which accommodates all RNA viruses with common phylogenetic lineages, except those utilizing reverse transcriptase. Note that large-scale evolutionary analysis of RNA viruses has still been controversial because only limited RdRP motifs are conserved (5, 6). However, some trends were indeed reproduced in other large-scale phylogenetic analyses of invertebrate RNA viruses (7).

Members of the kingdom Fungi host diverse RNA viruses (RNA mycoviruses) (8). Fungal dsRNA viruses with a diversified number of genomic segments (from 1 to 12) have been found (9), while non-, bi-, or trisegmented (−)RNA viruses have been reported (10–14). Fungal dsRNA viruses are generally encapsidated in spherical particles (9), while some fungal (−)RNA viruses are known to form nucleocapsid structures (11). For (+)RNA fungal viruses, nonsegmented viruses have been identified (8). These (+)RNA fungal viruses are largely capsidless and do not form typical virions, with some of them forming spherical, filamentous, or bacilliform particles (8). Recently, however, unusual fungal RNA viruses (polymycoviruses) with a mixed nature of (+)RNA, (−)RNA, and dsRNA viruses have been detected (15). Polymycoviruses, formerly tetramycoviruses, are multisegmented RNA viruses with a phylogenetic affinity for animal (+)RNA viruses, including walrus calicivirus and other caliciviruses within the expanded picornavirus-like superfamily (15, 16). Six published and four unpublished polymycoviruses with genomic sequences deposited in public databases have been nominated as members of a proposed dsRNA virus taxon, the genus “*Polymycovirus*” in the family “*Polymycoviridae*” in the realm *Riboviria*, according to the taxonomy proposal submitted to the ICTV (ICTV ratified [https://talk.ictvonline.org/files/proposals/taxonomy_proposals_fungal1/m/fung04/9294]). These 10 candidates were isolated from different fungal species but share four conserved genomic RNA segments, termed dsRNA1, dsRNA2, dsRNA3, and dsRNA4, that putatively encode an RdRP, a protein with unknown function (containing a transmembrane domain and a zinc-finger-like motif), a methyltransferase (MTR), and a proline-alanine-serine-rich protein (PASrp), respectively (15, 17–22). In addition to the four conserved genomic segments, some polymycoviruses have an additional one to four segments that share no similarity in their sequence to each other and to known protein sequences (17–20). RdRPs of most known dsRNA and (+)RNA viruses contain the “GDD” motif as the catalytic core residues (23, 24). However, in the putative RdRP of polymycoviruses, the catalytic residues are replaced with “GDNQ” (15, 17–20, 22), which is a hallmark of most (−)RNA mononegaviruses (23).

Other unusual properties of polymycoviruses include infectious entities (15, 19). The dsRNA form of polymycovirus genomic RNA is associated with the virally encoded PASrps (15, 17, 19) in at least two manners. The dsRNA of Aspergillus fumigatus tetramycovirus 1 (AfuTmV1), the exemplar strain of the proposed family *Polymycoviridae*, has been reported to be associated with PASrp in a colloidal form (15). On the other hand, the dsRNA of Colletotrichum camelliae filamentous virus 1 (CcFV1) has been shown to be encapsidated in filamentous particles, atypical of dsRNA viruses (19). Importantly, both colloidal and filamentous forms, which can be precipitated by ultracentrifugation (Ucfg) used for standard virion purification, are infectious when transfected into protoplasts of their original hosts (15, 19). More surprisingly, both viruses are also infectious as their deproteinized naked dsRNA (15, 19). The consensus for viral forms and infectious entities remains ambiguous for the majority of polymycoviruses.

Here, we characterize a unique polymyco-like virus from the ascomycetous fungus Fusarium oxysporum, one of the most destructive pathogens in agriculture (25, 26). Unique features of this novel virus, termed Hadaka virus 1 (HadV1), include an 11-segmented (+)RNA genome, including the 3 conserved segments encoding a putative RdRP (RNA1), a protein of unknown function (RNA2), and MTR (RNA3) of polymycoviruses but lacking the other conserved segment encoding PASrp. The Japanese term “Hadaka” literally means “naked.” Unlike typical polymycoviruses, HadV1 appears to be in a naked form and not to be pelleted by ultracentrifugation, implying a capsidless or naked nature of HadV1. This study revealed another unique lifestyle of a fungal RNA virus.

## RESULTS

### Novel polymycovirus-related dsRNA from Fusarium oxysporum.

A virus survey in a number of Pakistani fungal isolates belonging to various species was conducted using traditional dsRNA detection in 2016 to 2017 (27, 28). Among the dsRNA-positive fungal isolates, strain 7n was used in this study. For the identification of the fungal species, the internal transcribed spacer (ITS) region of fungal isolate 7n was sequenced. Isolate 7n was then identified as a strain of Fusarium oxysporum, which was further confirmed by sequencing of the intergenic spacer (IGS) region and the elongation factor 1 alpha gene (*ef1*α) (data not shown).

**FIG 1:**
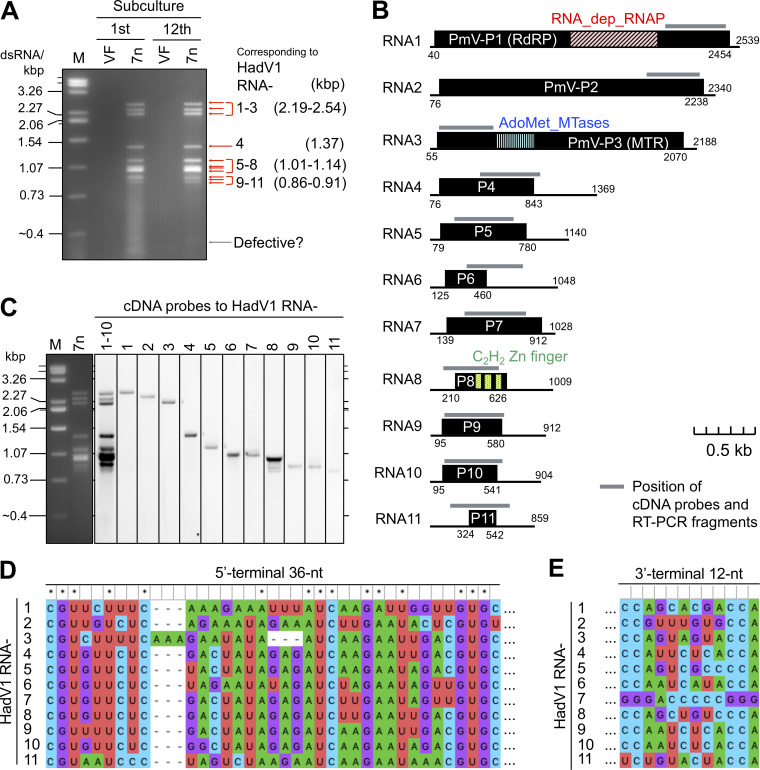
Molecular features of the HadV1 genome. (A) Electrophoretic profile of dsRNA purified from Fusarium oxysporum isolate 7n. The dsRNA was extracted by a cellulose affinity method and subsequently treated with DNase and S1 nuclease. Shown is the stability of the dsRNA profiles before and after subculturing of the hosts 12 times over 3  months. “VF” indicates the 7n-cf1 isolate, a virus-free conidial subisolate of 7n (see Fig. S6E in the supplemental material). Here, lane “M” indicates the dsRNA size marker (genomic dsRNA segments of mycoreovirus 1/S10ss) (66). (B) Schematic representation of the HadV1 genome. Each segment is identified with numbers in order from the longest to the shortest. The black boxes indicate hypothetical ORFs. The colored boxes in ORFs indicate positions of conserved domains or motifs (Table 2). RNA1, RNA2, and RNA3 encode proteins conserved in polymycoviruses, namely, PmV-P1, PmV-P2, and PmV-P3, respectively. Gray bars show positions of cDNA probes used in the subsequent panel (C) and RT-PCR fragments shown in Fig. 3A. (C) Assignment of the cDNA probes to the dsRNA segments extracted from F. oxysporum isolate 7n by Northern hybridization. (D) Multiple-sequence alignment of the 5′-terminal nucleotide sequences of the HadV1 genomic segments. The 5′-terminal 180-nt sequences of the genomic segments were aligned using ClustalW2. The 5′-terminal 36-nt sequences are shown. (E) Comparison of the 3′-terminal 12-nt sequences of the HadV1 genomic segments. For panels D and E, the aligned sequences were visualized with MEGA X.

The physical nature of the dsRNA purified from 7n was confirmed by its susceptibility to dsRNA-specific RNase III, and the sizes ranged from approximately 0.9 to 2.5 kbp, similar to the sizes of genomic segments of polymycoviruses (Table 1; see also Fig. S1 in the supplemental material). This dsRNA profile was stable through repeated subculture of the host fungal strain (Fig. 1A). The nucleotide sequence of the dsRNA was determined by next-generation sequencing (NGS) technology and subsequent reverse transcription-PCR (RT-PCR) and Sanger sequencing. By a BLASTX search (https://blast.ncbi.nlm.nih.gov/Blast.cgi), three of the NGS contigs showed low-identity (approximately 23 to 33% identity at the amino acid sequence level) but significant BLASTX hits to each dsRNA1-, dsRNA2-, and dsRNA3-encoded protein of the known polymycoviruses (Table 2). No NGS contigs showing significant similarity to polymycovirus dsRNA4-encoded PASrps were detected. So far, no polymycoviruses have been reported from F. oxysporum (Table 1). Thus, the polymyco-related virus found here was named Hadaka virus 1 (HadV1) because of its capsidless nature (see below), HadV1 was identified as a (+)RNA virus based on RdRP-based phylogeny (see below), as in the case of other capsidless (+)RNA viruses. Thus, the HadV1-derived dsRNA mentioned above and below was regarded as a replicative form.

**TABLE 1:** Known polymycoviruses and closely related viruses^*a*^

Full virus name	Virus abbreviation	Genome information		GenBank protein accession no.		Nominated^*b*^	Published^*c*^	Characterized^*d*^
		No. of deposited segments	Size range (bp)	RdRP	PASrp			
Aspergillus fumigatus tetramycovirus 1	AfuTmV1	4	1.1–2.4	CDP74618	CDP74621	✓	✓	✓
Beauveria bassiana polymycovirus 1	BbPmV1	4	1.3–2.4	CUS18595	CUS18598	✓	✓	✓
Botryosphaeria dothidea RNA virus 1	BdRV1	5	1.1–2.4	AKE49495	AKE49498	✓	✓	✓
Colletotrichum camelliae filamentous virus 1	CcFV1	8	1.0–2.4	ASV63092	ASV63095	✓	✓	✓
Fusarium redolens polymycovirus 1	FrPmV1	8	0.9–2.5	QDH44656	QDH44659	✓	✓	✓
Penicillium digitatum polymycovirus 1	PdPmV1	4	1.3–2.4	AVZ65983	AVZ65986	✓	✓	✓
Aspergillus spelaeus tetramycovirus 1	AspTmV1	4		AYP71805	AYP71806	✓		
Cladosporium cladosporioides virus 1	CcV1	5		AII80567	AII80570	✓		
Magnaporthe oryzae polymycovirus 1	MoPmV1	5		QAU09249	QAU09252	✓		
Penicillium brevicompactum tetramycovirus 1	PbTmV1	4		AYP71801	AYP71802	✓		
Aspergillus fumigatus polymycovirus 1^*e*^	AfuPmV1	4		AXE72937	AXE72940		✓	
Alternaria tenuissima virus		1		AJP08049^*g*^	Unidentified			
Beauveria bassiana polymycovirus 2	BbPmV2	3 (7)^*f*^		CUS18599	Unidentified		✓	
Beauveria bassiana polymycovirus 3	BbPmV3	3 (6)^*f*^		CUS18606^*g*^	Unidentified		✓	
Phaeoacremonium minimum tetramycovirus 1	PmTMV1	4		QDB74985	QDB74988		✓	
Plasmopara viticola-associated tetramycovirus 1		2		QHG11067	Unidentified			
Plasmopara viticola-associated tetramycovirus 2		3		QHG11070^*g*^	QHG11071			
Plasmopara viticola-associated tetramycovirus 3		1		QHG11072	Unidentified			
Plasmopara viticola-associated tetramycovirus 4		1		QHG11073^*g*^	Unidentified			
Plasmopara viticola-associated tetramycovirus 5		3		QHG11074	QHG11076			
Sclerotinia sclerotiorum tetramycovirus 1	SstRV1	3		AWY10945	Unidentified		✓	

a Virus isolates with an RdRP that shows more than 40% amino acid sequence identity to AfuTmV1 RdRP (see Table S1 at http://www.rib.okayama-u.ac.jp/pmi/Supplemental%20Material.html).

b Check marks indicate members nominated for the proposed genus *Polymycovirus* of the proposed family *Polymycoviridae*.

c Check marks indicate published virus strains. References can be found under the accession numbers.

d Check marks indicate characterized virus strains with complete genome information and some biological properties.

e AfuPmV1 is a variant of AfuTmV1.

f Numbers in parentheses indicate the number of hypothetical genome segments expected from gel electrophoresis.

g Only a partial sequence is available.

**TABLE 2:** Results of BLASTX searches with HadV1 genomic segments

Query	Hit^*a*^	Domain or motif	Provisional top 3 results (lowest E value)				
			Description	Query coverage (%)	Identity (%)	E value	GenBank accession no.
RNA1	Yes	RNA-dep RNAP^*b*^ (cd01699)	RNA-dependent RNA polymerase (Aspergillus fumigatus polymycovirus 1)	75	32.01	3e−84	YP_009551547.1
			RNA-dependent RNA polymerase (Aspergillus fumigatus tetramycovirus 1)	75	32.17	1e−83	CDP74618.1
			putative RNA-dependent RNA polymerase (Magnaporthe oryzae polymycovirus 1)	76	32.88	3e−81	QAU09249.1

RNA2	Yes	None	Hypothetical protein (Colletotrichum camelliae filamentous virus 1)	42	23.01	1e−06	ASV63093.1
			Hypothetical protein (Botryosphaeria dothidea virus 1)	38	23.39	5e−06	YP_009342447.1
			Putative aldehyde ferredoxin oxidoreductase (Botryosphaeria dothidea virus 1)	66	23.96	3e−05	ALZ41795.1

RNA3	Yes	AdoMet_MTase^*c*^ (cd02440)	Unnamed protein product (*Melampsora lini*)	64	24.80	2e−22	CAA45724.1
			Methyltransferase (Penicillium digitatum polymycovirus 1)	61	25.86	3e−14	YP_009551549.1
			Hypothetical protein (Magnaporthe oryzae polymycovirus 1)	60	26.70	2e−13	QAU09251.1

RNA4	No	None					

RNA5	No	None					

RNA6	No	None					

RNA7	No	None					

RNA8	Yes	C_2_H_2_ Zn finger	Zinc finger protein 26-like (Ixodes scapularis)	36	34.38	1e−07	XP_029825723.1
			Zinc finger protein 572-like (*Frankliniella occidentalis*)	23	38.75	1e−07	XP_026291381.1
			Gastrula zinc finger protein xLCGF3.1-like (*Boleophthalmus pectinirostris*)	37	32.65	2e−07	XP_020778374.1

RNA9	No	None					

RNA10	No	None					

RNA11	No	None					

a Presence or absence of hits by a BLASTX search of the nonredundant protein sequence (nr) database. Hits with a high E value (>1) were excluded.

b RNA-dependent RNA polymerase.

c *S*-adenosylmethionine-dependent methyltransferase.

10.1128/mBio.00450-20.1 FIG S1 Susceptibility of dsRNA purified from Fusarium oxysporum isolate 7n to RNase III. The dsRNA fractions from HadV1-infected (7n) and uninfected (VF) fungal isolates (see Fig. S6E in the supplemental material) were treated with or without ShortCut RNase III. Download FIG S1, JPG file, 0.1 MB. Copyright © 2020 Sato et al. 2020 Sato et al. This content is distributed under the terms of the Creative Commons Attribution 4.0 International license.

### Full-length genome sequence and molecular features of HadV1.

Agarose gel electrophoresis of the 7n dsRNA showed at least nine distinguishable bands (Fig. 1A and Fig. S1). To determine whether these dsRNA elements were from HadV1 alone or from multiple RNA viruses, we prepared cDNA libraries of the 7n dsRNA by a method for random cDNA synthesis and 3′ RNA ligase-mediated rapid amplification of cDNA ends (3′ RLM-RACE). Approximately 300 obtained cDNA clones were sequenced by Sanger’s method, and a total of 11 contigs, including the three above-mentioned potential viral segments, were obtained. The positive strands of the 11 fully sequenced dsRNA segments were designated RNA1 to RNA11 in order of nucleotide length from the longest (2,539 nucleotides [nt]) to the shortest (859 nt) (Fig. 1B). A dsRNA band slightly shorter than 400 bp appeared to be a mixture of defective molecules of some genomic segments, as shown by cloning and sequencing of the band (data not shown). This was supported by the observation that the relative density of this short (<400-bp) band varied depending on the subcultures of the host (see Fig. 1, 3, 5, and 6; see also Fig. S1). By Northern hybridization analysis, cDNA probes for each of the 11 segments were individually assigned to all the visible dsRNA bands of the expected sizes (Fig. 1C). Multiple-sequence alignment revealed that the positive strands of the 11 dsRNA segments shared the common 5′-terminal nucleotide sequence “CGU” followed by a moderately conserved sequence (Fig. 1D). The 11 segments, except for RNA7, also share the common 3′-terminal nucleotide sequence “CC(A)” (here, the nucleotide “A” at the 3′ terminus was contained in only some RACE clones) (Fig. 1E). These conserved terminal sequences of the 11 segments support the hypothesis that all 11 RNA segments represent the genome of HadV1 (Fig. 1B).

The putative proteins encoded by the HadV1 genome were subjected to a BLASTX search. As mentioned above, HadV1 RNA1, RNA2, and RNA3 were homologous to dsRNA1, dsRNA2, and dsRNA3 of polymycoviruses, respectively (Table 2). The HadV1 RNA8 hypothetical protein contained C_2_H_2_-type zinc finger motifs and showed similarity to zinc finger proteins from various organisms (Table 2 and Fig. 1B). No other segments (RNA4 to RNA7 and RNA9 to RNA11) showed significant similarity to known sequences (Table 2). For these segments, the longest hypothetical open reading frame (ORF) on positive strands is indicated in Fig. 1A and Fig. S2A. As with the results of NGS analysis, none of the 11 segments showed similarity to polymycovirus dsRNA4 encoding a PASrp.

10.1128/mBio.00450-20.2 FIG S2 Comparison between the putative proteins encoded on the HadV1 genome and the putative polymycovirus PASrps. (A) Comparison of the HadV1 genomic segments with the PASrp-encoding segments of 10 polymycoviruses. RdRP, MTR, and ZF denote RNA-dependent RNA polymerase, a putative methyltransferase, and a zinc-finger-like protein, respectively. The black boxes indicate hypothetical ORFs. The values shown on the right indicate the estimated molecular mass (kilodaltons) of each hypothetical protein. (B) Amino acid compositions of the putative proteins. GenBank accession numbers for the amino acid sequences of polymycovirus PASrps are listed in Table 1. The amino acid compositions were calculated by using the ProtParam tool on the ExPASy website (http://www.expasy.org). Download FIG S2, JPG file, 0.6 MB. Copyright © 2020 Sato et al. 2020 Sato et al. This content is distributed under the terms of the Creative Commons Attribution 4.0 International license.

Because PASrp is a hallmark of polymycoviruses, further attempts were made to search HadV1 proteins for PASrp-like proteins. Some genomic segments of HadV1, such as S4 and S5, have coding capacities similar to those of the PASrp-encoding segments of polymycoviruses (Fig. S2A). The proteins hypothetically encoded by HadV1 RNA4, RNA5, RNA7, and RNA9 (named P4, P5, P7, and P9) contained relatively more PAS (proline, alanine, and serine) residues (20 to 23%) than the others encoded by HadV1 (Fig. S2B). However, this ratio was still lower than that of PASrps of the known polymycoviruses (24 to 32%) (Fig. S2B). Moreover, the deduced amino acid sequences of all the HadV1 proteins P4, P5, P7, and P9 were poorly aligned to those of PASrps encoded by polymycoviruses, although the known polymycovirus PASrps were better conserved (Fig. S3). Thus, HadV1 seemed to lack PASrps conserved in the other polymycoviruses.

10.1128/mBio.00450-20.3 FIG S3 Multiple-sequence alignment of the deduced amino acid sequences of the HadV1 hypothetical proteins (P4, P5, P7, and P9) and the polymycovirus PASrps using PSI-Coffee. The black arrow indicates scores of consistency within the alignment. Download FIG S3, JPG file, 1.4 MB. Copyright © 2020 Sato et al. 2020 Sato et al. This content is distributed under the terms of the Creative Commons Attribution 4.0 International license.

### Phylogenetic relationships between HadV1 and known polymycoviruses.

Based on a BLASTP search, the RdRP sequences of HadV1 and the proposed polymycoviruses, except for AfuTmV1, showed similarity exclusively to RdRPs of polymycoviruses and related viruses listed in Table 1 (see also Table S1 at http://www.rib.okayama-u.ac.jp/pmi/Supplemental%20Material.html). AfuTmV1 shared BLASTP-detectable RdRP sequences with only some vesiviruses in the family *Caliciviridae* in addition to polymycoviruses (see Table S1 at the URL mentioned above). These results suggested that HadV1 and polymycoviruses have very distant phylogenetic relationships to known viruses except for vesiviruses.

**FIG 2:**
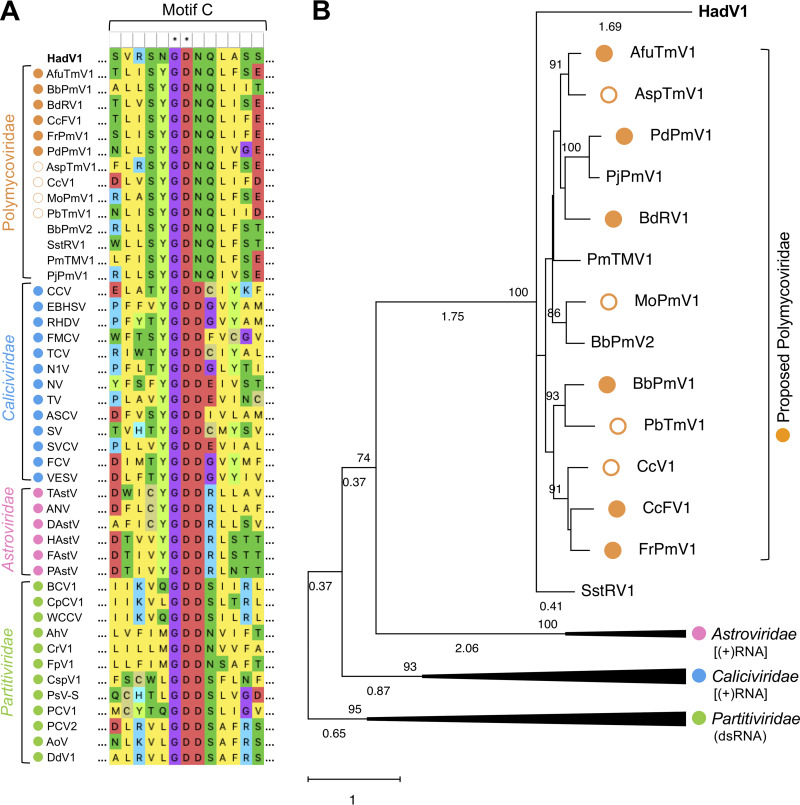
Phylogenetic analysis of RdRPs of HadV1, polymycoviruses, and some expanded members of the picorna-like virus superfamily (Table 1; see also Table S2 at http://www.rib.okayama-u.ac.jp/pmi/Supplemental%20Material.html for the viruses included in the analysis). The closed and open orange circles indicate published and unpublished members of the proposed family *Polymycoviridae*, respectively. Viruses that are closely related to polymycoviruses but not assigned to any taxa (Table 1) are indicated without orange circles. The cyan, pink, and yellow-green filled circles indicate caliciviruses, astroviruses, and partitiviruses, respectively. (A) Alignment of conserved RdRP motif C sequences. The aligned sequences were visualized using MEGA X. (B) Phylogenetic tree based on the RdRP alignment. The maximum likelihood tree (model LG+G+I) was constructed in MEGA X. The values at the upper left of the branches indicate the bootstrap probability with 500 replicates. Bootstrap values of <70% are hidden. The scale bar and branch length indicate the number of amino acid differences per site. The values under the branches indicate the branch length. Branch lengths of <0.32 are hidden. The clades of the families *Caliciviridae*, *Astroviridae*, and *Partitiviridae* are compressed. The original tree is shown in Fig. S5 in the supplemental material.

Previous phylogenetic analyses based on RdRP implied that polymycoviruses were classified into the same clade as (+)RNA viruses in the families *Caliciviridae* and *Astroviridae* rather than with dsRNA viruses in the families *Partitiviridae* and *Amalgaviridae* (18). Thus, we analyzed RdRP-based phylogenetic relationships between HadV1 and the other polymycoviruses (Table 1) or members of the families *Caliciviridae*, *Astroviridae*, and *Partitiviridae* in the picornavirus-like supergroup (see Table S2 at the URL mentioned above). The multiple-sequence alignment showed that HadV1 RdRP as well as RdRPs of polymycoviruses possessed several domains conserved in these picornavirus-like supergroup members (Fig. S4; see also Text S1 at the URL mentioned above). HadV1 as well as polymycoviruses, however, had “GDNQ” as the hypothetical catalytic core residues in RdRP motif C instead of “GDD” (Fig. 2A). A phylogenetic tree constructed based on the alignment (see Texts S1 and S2 at the URL mentioned above) showed that HadV1 was placed into the same clade as the known polymycoviruses (Fig. 2B and Fig. S5). However, HadV1 seemed more distantly related to these polymycoviruses than they were from one another (Fig. 2B and Fig. S5). As previously inferred (18), HadV1 and polymycoviruses appeared to be phylogenetically classified into the same clade as (+)RNA astroviruses, although the branch probability was not extremely high (74%) (Fig. 2B and Fig. S5).

10.1128/mBio.00450-20.4 FIG S4 Part of the multiple-sequence alignment of the RdRP sequences of HadV1 and polymycoviruses. The filled and unfilled orange circles indicate published and unpublished polymycoviruses, respectively. Viruses that are closely related to polymycoviruses but not assigned to any taxa (Table 1) are indicated without orange circles. The pink, cyan, and yellow-green circles refer to (+)RNA astroviruses, (+)RNA caliciviruses, and dsRNA partitiviruses, respectively. The aligned sequences around motifs A and B of RdRP are shown. The entire alignment is shown in Texts S1 and S2 at http://www.rib.okayama-u.ac.jp/pmi/Supplemental%20Material.html. The alignment was visualized with MEGA X. Download FIG S4, JPG file, 2.0 MB. Copyright © 2020 Sato et al. 2020 Sato et al. This content is distributed under the terms of the Creative Commons Attribution 4.0 International license.

10.1128/mBio.00450-20.5 FIG S5 Maximum likelihood phylogenetic tree based on RdRP amino acid sequences. This tree shows all viruses hidden in Fig. 2B. See the legend of Fig. S4 in the supplemental material for an explanation of the colored circles. The values at the upper left of the branches indicate bootstrap probability with 500 replicates. Bootstrap values of <70% are hidden. The scale bar and branch length indicate the number of amino acid differences per site. Download FIG S5, JPG file, 0.3 MB. Copyright © 2020 Sato et al. 2020 Sato et al. This content is distributed under the terms of the Creative Commons Attribution 4.0 International license.

Taken together, HadV1 as well as phylogenetically closely related polymycoviruses seem to show a higher phylogenetic affinity for (+)RNA viruses than for dsRNA viruses.

### Vertical transmission of HadV1 through conidia and its phenotypic effects on the host fungus.

**FIG 3:**
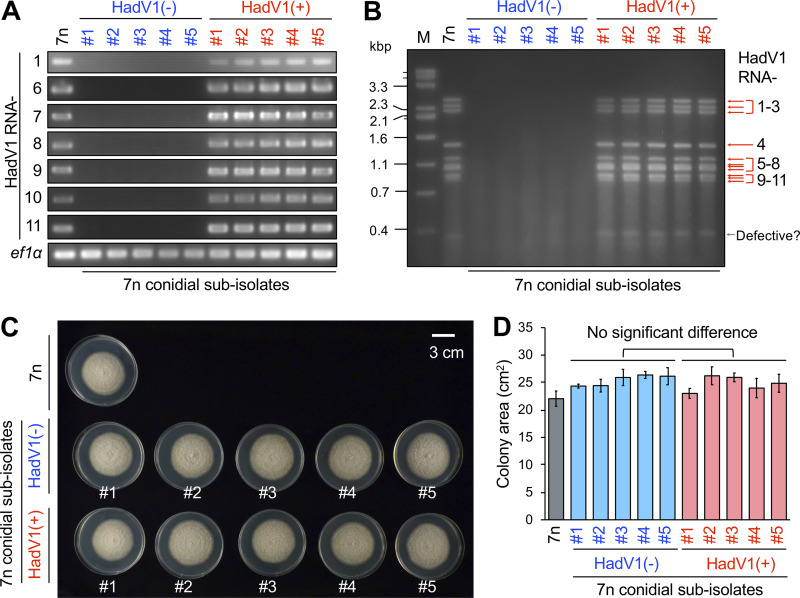
HadV1 transmission to single conidial subisolates of F. oxysporum isolate 7n and growth comparison between virus-free and -infected colonies. (A) Detection of HadV1 genomic RNA segments by RT-PCR using purified ssRNA as a template. RNA1 and other genomic segments, which are difficult to separate on an agarose gel, were targeted by RT-PCR. The *ef1*α gene of F. oxysporum was employed as an internal control. HadV1(−) and HadV1(+) were confirmed to be virus negative and virus positive by direct colony one-step RT-PCR (see Fig. S6B in the supplemental material). The five subisolates representing each of the HadV1(−) and HadV1(+) groups were selected from conidia harvested from young (2- to 4-day-old) mycelia of the 7n strain. (B) Electrophoretic gel profile of dsRNA (viral replicative form) from HadV1(−) and HadV1(+) subisolates. (C) Colony morphology of 6-day-old cultures of HadV1(−) and HadV1(+) subisolates on PDA medium. (D) Areas of 6-day-old PDA cultures of HadV1(−) and HadV1(+) subisolates. The colony areas shown in Fig. 1C were measured using ImageJ (http://imagej.nih.gov/ij/). The mean values ± standard deviations (SD) from five replicates are shown. Differences between the HadV1(−) and HadV1(+) subisolates were statistically analyzed by two-way analysis of variance (ANOVA) (Bartlett’s test) followed by a *t* test (*n *= 5; *P < *0.05) using R version 3.5.2 (https://www.r-project.org).

The 11 dsRNA segments were maintained during repeated subculture of single HadV1-infected isolates. We tested by one-step RT-PCR how frequently HadV1 was vertically transmitted via asexual spores (Fig. S6A) present on old and young host fungal colonies. This assay revealed that the rate of transmission of HadV1 to conidia varied depending on the age of the mycelia from which conidia were taken (Fig. S6B). HadV1 transmission rates in conidia derived from younger (2- to 4-day-old) mycelia were much higher (89 to 93%) than in those taken from older (10- to 12-day-old) mycelia (15 to 20%) (Fig. S6C). This phenomenon evoked the previous observation that some dsRNA viruses were distributed unevenly; growing mycelia at the edge of an ascomycetous fungus colony contained more viral dsRNA than those in the central area (29). Of the conidial isolates derived from younger mycelia, five representatives each of the HadV1-infected [HadV1(+)] and HadV1-free [HadV1(−)] groups (Fig. 3A) were chosen for analysis of the electrophoretic profile of dsRNA. All five representatives of HadV1(−) subisolates showed no dsRNA bands, whereas all five HadV1(+) subisolates exhibited a dsRNA banding pattern indistinguishable from that of the original 7n strain (Fig. 3B). The apparent all-or-none transmission of the 11 segments was observed for other conidial subisolates derived from both young and old mycelia (Fig. S6D and E). The presence of HadV1 RNA segments of similar sizes (RNA6 to RNA11) in HadV1(+) conidial subisolates was confirmed by RT-PCR (Fig. 3A). These data strongly support the above-mentioned notion that all 11 dsRNA elements are the genomic segments of the single virus HadV1.

The preparation of isogenic HadV1(−) and HadV1(+) fungal subisolates allowed us to investigate HadV1 effects on the host fungus. Colony size and morphology were indistinguishable between the HadV1(−) and HadV1(+) subisolates, suggesting asymptomatic infection of F. oxysporum by HadV1 on a nutrient-rich medium (Fig. 3C and D and Fig. S7).

10.1128/mBio.00450-20.6FIG S6All-or-none transmission of viral dsRNA segments to single conidial subisolates of F. oxysporum isolate 7n. (A) Microscopic observation of conidia harvested from isolate 7n. (B) Detection of HadV1 RNA1 (RdRP-encoding segment) in 7n single conidial subisolates by direct colony one-step RT-PCR. Conidia taken from younger (2- to 4-day-old) (top) or older (10- to 12-day-old) (bottom) mycelia were tested. The *ef1*α gene of F. oxysporum was employed as a control for RT-PCR. (C) Frequency of HadV1 vertical transmission through conidiation. Single conidial subisolates were prepared from young (2- to 4-day-old) and old (10- to 12-day-old) colonies and tested for the presence of virus. HadV1(−) and HadV1(+) indicate the absence and presence of virus, respectively. The assay was repeated twice (Rep1 and Rep2). (D and E) Electrophoretic gel profile of dsRNA fractions from 7n single conidial subisolates. HadV1(−) (7n-cf*n*) and HadV1(+) (7n-cv*n*) subisolates were obtained from old (D) or young (E) colonies. All the conidial subisolates here are different from the subisolates used in Fig. 3. The subisolates designated 7n-cf*n* and 7n-cv*n* in panel E were used in Fig. S7 in the supplemental material. A different “*n*” represents a different independent subisolate. Download FIG S6, JPG file, 0.5 MB.Copyright © 2020 Sato et al.2020Sato et al.This content is distributed under the terms of the Creative Commons Attribution 4.0 International license.

10.1128/mBio.00450-20.7 FIG S7 Comparison of mycelial growth between HadV1-free (−) and HadV1-infected (+) single conidial subisolates of F. oxysporum isolate 7n. (A) Colony morphology of representative HadV1(+) and HadV1(−) cultures on PDA medium. The conidial subisolates described in the legend of Fig. S6E in the supplemental material were grown on PDA for 5 days and photographed. (B) Mycelial area estimated with ImageJ. The mean values ± SD for six replicates are shown. Statistical differences between HadV1(−) and HadV1(+) subisolates were analyzed by two-way ANOVA (Bartlett’s test) followed by Welch’s *t* test (*n *= 6; *P < *0.01) using R software. Note that mycelial growth was slightly faster than that in Fig. 3C and D due to seasonal effects on the laboratory bench. Download FIG S7, JPG file, 0.5 MB. Copyright © 2020 Sato et al. 2020 Sato et al. This content is distributed under the terms of the Creative Commons Attribution 4.0 International license.

### Attempts to purify HadV1 particles by a conventional method.

**FIG 4:**
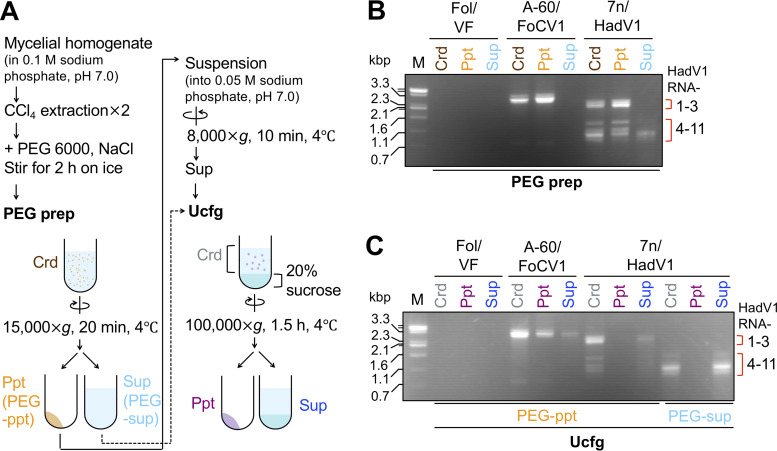
Attempts to purify HadV1 particles by a standard method. (A) Schematic representation of the fractionation of virus particles by PEG precipitation (PEG prep) and ultracentrifugation (Ucfg). Crd, Ppt, and Sup denote the crude extract, pellet, and supernatant fractions, respectively. (B) Electrophoretic gel profile of dsRNA extracted from the fractions before (Crd) and after (Ppt and Sup) PEG prep. (C) Agarose gel pattern of dsRNA in the fractions before (Crd) and after (Ppt and Sup) Ucfg. In addition to HadV1-infected 7n (7n/HadV1), a virus-free Japanese isolate of F. oxysporum f. sp. *lycopersici* (Fol/VF) and a Pakistani isolate of F. oxysporum infected by an encapsidated dsRNA chrysovirus (Fusarium oxysporum chrysovirus 1 [FoCV1]) were used as references in panels B and C.

To analyze a possible HadV1 form in infected mycelia, we first attempted to purify virus particles by a conventional method that had been applied to a variety of encapsidated mycoviruses (27, 28, 30–32). In this method, virus particles are typically condensed by two steps: precipitation with polyethylene glycol (PEG prep) in the presence of sodium chloride (NaCl), followed by ultracentrifugation (Ucfg) (approximately 100,000 × *g* for 1.5 h) of resuspended particles on a 20% sucrose cushion (Fig. 4A). As a control for this experiment, we used an encapsidated dsRNA virus, Fusarium oxysporum chrysovirus 1 (FoCV1) (a novel virus of the genus *Alphachrysovirus*), harbored in an isolate (A-60) of F. oxysporum. Chrysoviruses have multipartite dsRNA genomes enclosed in icosahedral capsids (33).

At first, we examined whether the viral dsRNA segments were coprecipitated with PEG-NaCl. After PEG prep, all the genomic segments of the chrysovirus (FoCV1) were detected in the pellet but not in the supernatant (Fig. 4B). However, some portions of the short HadV1-derived dsRNA segments remained in the supernatant, although most of the HadV1 segments were precipitated (Fig. 4B). These results eliminate the possibility that dsRNA segments of HadV1 are all packaged in single particles.

Next, we examined where the dsRNA segments were fractionated after Ucfg. For HadV1, the resuspended PEG precipitant (PEG-ppt) and the supernatant after PEG-NaCl-mediated precipitation (PEG-sup) were separately ultracentrifuged (Fig. 4A, arrows with solid and dotted lines, respectively). After Ucfg, the majority of the FoCV1 genomic dsRNA was detected in the pellets (Fig. 4C), which supported that encapsidated particles were precipitable under this condition. In contrast, after the same ultracentrifugation step, HadV1 dsRNA in both PEG-ppt and PEG-sup remained in the supernatant (Fig. 4C). HadV1 single-stranded RNA (ssRNA) was also not detected in the pellet after Ucfg when the total nucleic acids were extracted by phenol-chloroform followed by ethanol precipitation (data not shown). Thus, neither dsRNA nor ssRNA of HadV1 appears to be encapsidated. These results were similar to those from a previous attempt to purify a capsidless (+)RNA fusarivirus from an ascomycetous fungus (34).

**FIG 5:**
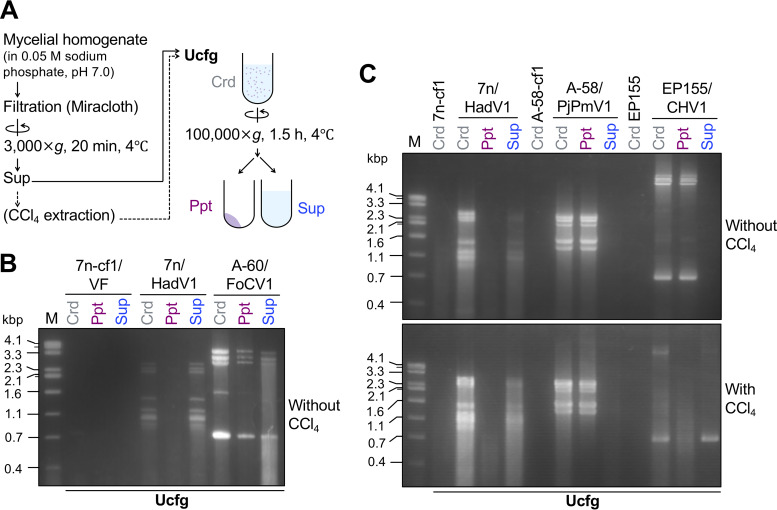
Analysis of the HadV1 form by a simple ultracentrifugation (Ucfg) method with or without clarification by the organic solvent CCl_4_. (A) Schematic representation of the purification procedure. (B and C) Agarose gel profiles of viral dsRNA extracted from three fractions, crude extracts (Crd) before Ucfg and pellets (Ppt) and supernatants (Sup) after Ucfg. (B) Comparison of HadV1 with an encapsidated dsRNA chrysovirus, FoCV1. Three fungal strains, a virus-free conidial subisolate of 7n (7n-cf1/VF) (see Fig. S6E in the supplemental material), HadV1-infected 7n (7n/HadV1), and a FoCV1-infected isolate (A-60/FoCV1) of F. oxysporum, were used. The CCl_4_ clarification step was omitted in this experiment. (C) Comparison of HadV1 with a typical polymycovirus (Penicillium janthinellum polymycovirus 1 [PjPmV1]) and an established capsidless hypovirus (Cryphonectria hypovirus 1 [CHV1]). Besides HadV1-infected 7n, two additional fungal strains, PjPmV1-infected *Penicillium janthinellum* isolate A-58 and CHV1-infected *C. parasitica* EP155, were used for fractionation with and without CCl_4_ clarification. Isogenic virus-free strains of the respective three strains, 7n-cf1, A-58-cf1, and EP155, were used to obtain their Crd fractions.

To further confirm the capsidless nature of HadV1, we utilized a milder purification procedure. The above-described virus purification protocol included a clarification step using an organic solvent for initial extraction. In contrast, particles or colloidal forms of polymycoviruses have been extracted without organic solvents (15, 17, 19). It has been shown that these polymycoviruses are precipitable by Ucfg (approximately 100,000 to 110,000 × *g* for 1 to 2 h). Thus, we next examined whether presumed HadV1 particles were pelleted by Ucfg of mycelial extracts that were obtained without organic solvent extraction (Fig. 5A). After Ucfg, HadV1-derived dsRNA appeared in the supernatant, but not in the pellet, whereas the genomic dsRNA of the encapsidated chrysovirus (FoCV1) was detected in the precipitated fraction (Fig. 5B). Also, no HadV1-derived ssRNA appeared in the pellet after Ucfg (Fig. S8). Importantly, dsRNA of a novel polymycovirus, Penicillium janthinellum polymycovirus 1 (PjPmV1), from an isolate (A-58) of Penicillium janthinellum could be pelleted when tested in parallel in this study (Fig. 5C, top). PjPmV1 shows greater sequence similarity to typical polymycoviruses (Fig. 2B) and has a PASrp-encoding genomic segment (Y. Sato and N. Suzuki, unpublished results). dsRNA of a well-characterized capsidless (+)RNA virus, Cryphonectria hypovirus 1 (CHV1, family *Hypoviridae*), from Cryphonectria parasitica was also pelleted (Fig. 5C, top). The replicative form of CHV1 is enclosed by cytoplasmic lipid vesicles (35, 36). Thus, when mycelial crude extracts were clarified with an organic solvent, CCl_4_ (Fig. 5A), dsRNA of CHV1 as well as dsRNA of HadV1 remained in the supernatant after Ucfg (Fig. 5C, bottom). In contrast, PjPmV1 particles, once subjected to CCl_4_ extraction, were still pelleted by Ucfg (Fig. 5C, bottom).

10.1128/mBio.00450-20.8 FIG S8 Fractionation of HadV1-derived RNA via ultracentrifugation (Ucfg). The experimental procedure is described in the legend of Fig. 5A. Crd, Ppt, and Sup denote crude extract, pellet, and supernatant fractions, respectively. An electrophoretic gel profile of ssRNA-enriched total nucleic acids precipitated with 2 M LiCl is shown. An HadV1-free isolate (7n-cf1/VF) and a fungal strain (A-60) infected with an encapsidated chrysovirus, FoCV1, were used as references. Download FIG S8, JPG file, 0.1 MB. Copyright © 2020 Sato et al. 2020 Sato et al. This content is distributed under the terms of the Creative Commons Attribution 4.0 International license.

These combined results showed that HadV1 has an extremely low sedimentation coefficient compared to the previously characterized polymycoviruses, the membrane vesicle-associated capsidless hypoviruses, and the encapsidated viruses, suggesting a unique capsidless nature of HadV1.

### *In vitro* susceptibility of HadV1 RNAs to RNase A.

**FIG 6:**
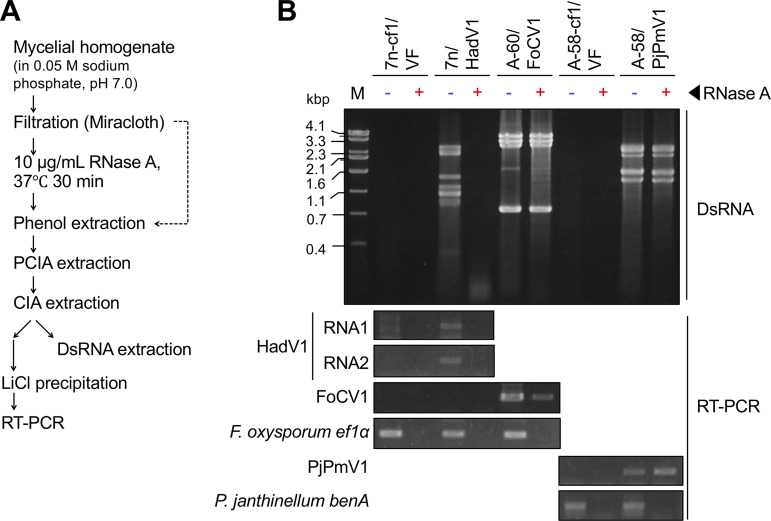
RNase A treatment of mycelial extracts containing viral RNAs. Three F. oxysporum strains (a virus-free isolate [7n-cf1/VF], a HadV1-infected isolate [7n/HadV1], and a FoCV1-infected isolate [A-60/FoCV1]) and two *P. janthinellum* strains (a virus-free isolate [A-58-cf1/VF] and a PjPmV1-infected isolate [A-58/cf1]) were used. (A) Flow of the experiment. (B) Susceptibility of viral RNAs to RNase A. Electrophoretic profiles of viral dsRNA (top) and RT-PCR products from total RNA (bottom) detected before and after RNase A treatment are shown. Host mRNAs (F. oxysporum *ef1*α and *P. janthinellum benA*) were targeted in parallel by RT-PCR.

To further validate the capsidless nature of HadV1, mycelial homogenates filtered through Miracloth were treated with RNase A under low-salt conditions that would digest both ssRNA and dsRNA (Fig. 6A). After RNase A treatment, the HadV1 dsRNAs appeared to be completely digested, while dsRNA of FoCV1 (an encapsidated dsRNA virus) and PjPmV1 (a polymycovirus associated with PASrp) remained intact (Fig. 6B, top). We further examined whether HadV1 ssRNAs were accessible by RNase in mycelial extracts by RT-PCR (Fig. 6A). RT-PCR products of host mRNAs (*ef1*α and *benA*), which could be observed in the RNase A-untreated samples, were undetectable in all the RNase-treated samples (Fig. 6B, bottom), validating this RNase A assay. Similarly, no RT-PCR product of HadV1 RNAs (RNA1 and RNA2) was detected in the RNase-treated sample (Fig. 6B, bottom). In contrast, FoCV1- and PjPmV1-derived fragments were amplified irrespective of whether the mycelial homogenates were treated with RNase A (Fig. 6B, bottom). The RT-PCR fragments of FoCV1 and PjPmV1, which were amplified in the RNase A-treated extracts, appeared to derive from a tiny amount of viral dsRNA copurified with LiCl-precipitated ssRNA fractions.

Collectively, these results further support that HadV1-derived RNAs exist in a capsidless form, which is distinct from the polymycovirus PjPmV1 and the chrysovirus FoCV1.

## DISCUSSION

Here, we identified and characterized a novel polymyco-related virus, HadV1, harbored in a Pakistani isolate, 7n, of the phytopathogenic ascomycete F. oxysporum. HadV1 had an 11-segmented genomic (+)RNA, 3 segments of which showed significant similarity to the conserved segments (dsRNA1, dsRNA2, and dsRNA3) of polymycoviruses (Fig. 1 to 3 and Table 2). Unlike the known polymycoviruses, HadV1 lacked genomic segments encoding typical PASrps (Table 2; see also Fig. S2 and S3 in the supplemental material). Possibly due to that, HadV1 was not pelleted by standard ultracentrifugation by which encapsidated RNA viruses and PASrp-associated polymycoviruses could be pelleted (Fig. 4 and 5). HadV1 RNAs in mycelial homogenates were susceptible to RNase A *in vitro* (Fig. 6). These results strongly suggest that HadV1 is capsidless, although we cannot rule out the possibility that HadV1 has atypical protein coats that are extremely vulnerable to the purification steps. Attempts to transfect virus-free F. oxysporum with HadV1-derived dsRNA were unsuccessful (data not shown). Thus, the infectivity of purified viral dsRNA, which was reported previously for two polymycoviruses (15, 17), may not be the case for HadV1. Thus, this study suggests a potential unique lifestyle or infection entity of a polymyco-like virus, HadV1, which can be distinct from the lifestyles of known polymycoviruses, capsidless (+)RNA hypoviruses, and encapsidated dsRNA viruses (Fig. 4 and 5).

Encapsidated viruses in the realm *Riboviria* can be simply classified according to their forms of encapsidated genomic RNA. How about capsidless viruses? Capsidless RNA viruses such as hypoviruses (family *Hypoviridae*) and endornaviruses (family *Endornaviridae*) had historically been classified as dsRNA viruses by the ICTV until recently because their dsRNA molecules as a replicative form accumulate in large amounts in infected host cells and are readily detectable. However, the ICTV has reclassified them as (+)RNA viruses according to RdRP- and/or RNA helicase-based phylogeny (37, 38). By analogy, HadV1 should be regarded as a (+)RNA virus rather than a dsRNA virus because HadV1 and polymycoviruses show a higher phylogenetic affinity for (+)RNA viruses such as caliciviruses and astroviruses than for dsRNA viruses (Fig. 2B and Fig. S5; see also Table S1 at http://www.rib.okayama-u.ac.jp/pmi/Supplemental%20Material.html). Note that the phylogenetic positions of members of branch II proposed by Wolf et al. (4), along with HadV1 and polymycoviruses, remain ambiguous due to the absence of closely related viruses that are expected to fill the intergroup gaps.

The number of segments, 11, of HadV1 is the largest in RNA viruses, excluding 11- or 12-segmented monoparticulate dsRNA reoviruses forming multilayered icosahedral particles. Polymycoviruses are diverse in the number of genomic segments, as in the case of mycoreoviruses (11 or 12 dsRNA genome segments) (39–41) and chrysoviruses (3 to 7 dsRNA genome segments) (33). Compared to other polymycoviruses (Table 1), HadV1 has the largest number of genomic segments. Spontaneous losses of dsRNA genomic segments during subculturing of infected fungal colonies have been reported for mycoreovirus 3 (42), Magnaporthe oryzae chrysovirus 1 (43), and Rosellinia necatrix megabirnavirus 2 (a bipartite dsRNA virus of the family *Megabirnaviridae*) (44). Some HadV1 genomic segments could be dispensable under laboratory conditions, and experiments aimed at testing this possibility are under way. However, a set of 11 HadV1 genomic segments was transmitted in an all-or-none fashion during asexual sporulation and repeated subculturing of an infected colony (Fig. 1 and 3 and Fig. S6). There appears to be a mechanism that allows efficient transmission and maintenance of the HadV1 genomic segments.

Generally, viral capsids seem to play multiple roles, such as those in the protection of genomic RNA, efficient transmission, and cell-to-cell and intertissue movements. Nevertheless, capsidless RNA viruses have been frequently found in fungi, possibly because they could complete their systemic infection and transmission without extracellular phases (8, 45). All well-characterized naked RNA viruses, such as the members of the families *Hypoviridae*, *Narnaviridae*, and *Endornaviridae*, have nonsegmented (+)RNA genomes (37, 38, 46). dsRNAs (replicative form) of hypoviruses and endornaviruses are considered to be encased in cytoplasmic membranous vesicles with active RdRPs (35, 36, 47–50), while narnavirus genomic RNA is associated with its RdRP in the cytosol (51). In fungi, RNA silencing (or RNA interference) works as a primary antiviral defense against which RNA viruses need to protect their mRNA, replicative intermediates, and replicative form (52, 53). Antiviral RNA silencing is substantiated in several filamentous fungi (52–54), including a *Fusarium* species (55). It is known that hypoviruses are targeted by antiviral RNA silencing, while they encode RNA silencing suppressors (52, 56, 57). The budding yeast Saccharomyces cerevisiae, hosting the well-studied narnaviruses Saccharomyces cerevisiae virus 20S (ScV20S) and ScV23S, lacks antiviral RNA silencing (58), although it has different layers of antiviral defense (59, 60). Recently, another capsidless lifestyle was proposed for a nonsegmented (+)RNA mycovirus termed yado-kari virus 1 (YkV1). Capsidless YkV1 hijacks capsids from an unrelated dsRNA virus, yado-nushi virus 1 (YnV1), possibly as the site for replication, and depends on YnV1 for encapsidation (32). Since polymycoviruses and yadokariviruses are commonly related to caliciviruses, a possible evolutionary relevance of their capsidless lifestyles was assumed (61), although they appear to have evolved different survival strategies. It remains unknown whether these related capsidless viruses are targeted by antiviral RNA silencing or whether they encode RNA silencing suppressors. Studies aimed at addressing these questions as well as defining the infectious entity of HadV1 are under way.

## MATERIALS AND METHODS

### Fungal strains and growth conditions.

The F. oxysporum strains described below were subcultured on Difco potato dextrose agar (PDA) medium (Becton, Dickinson and Co.). The PDA cultures were placed on a laboratory bench near windows at room temperature (23°C to 27°C). For subculture, a 1-mm^3^ fresh mycelial plug was transferred to new PDA in flat petri dishes (9 cm in diameter) every 7 to 10 days. For a long-term stock, mycelial plugs were stored in 10% (vol/vol) glycerol at −80°C.

The F. oxysporum isolate 7n was collected from a tomato in a vegetable market in Pakistan. Isolate 7n was identified as F. oxysporum by sequencing of PCR products of the ITS amplified with primers ITS1 and ITS4 (62), the IGS amplified with primers FIGS11 and FIGS12 (63), and *ef1*α amplified with primers EF-1 and EF-2 (64) (see also Table S3 at http://www.rib.okayama-u.ac.jp/pmi/Supplemental%20Material.html). Virus-free isogenic subisolates of isolate 7n were obtained by single-conidium isolation as described below.

For virus purification experiments, a Pakistani isolate (A-60) of F. oxysporum, infected with a novel chrysovirus (FoCV1) (A. Jamal and N. Suzuki, unpublished data), was used as a control host of an encapsidated virus. In addition, a Japanese strain (9859-1) of F. oxysporum f. sp. *lycopersici* race 2, a generous gift from Masashi Matsusaki of the Aichi Agricultural Research Center, was used in several experiments as a virus-free control (Fig. 4). A Pakistani isolate (A-58) of P. janthinellum was used as a control infected by a typical polymycovirus (Penicillium janthinellum polymycovirus 1 [PjPmV1]) (Sato and Suzuki, unpublished). As a virus-free isogenic control for A-58, one of its conidial subisolates (A-58-cf1) was used. A standard strain (EP155) of C. parasitica infected with the exemplar strain of hypovirus, CHV1, was also used (65).

### Extraction, gel electrophoresis, and Northern hybridization of dsRNA.

dsRNA was extracted from mycelia cultured on PDA-cellophane for 2 days by cellulose column chromatography as previously described (27). The dsRNA extracts were further treated with RQ1 DNase (Promega Corp.) and S1 nuclease (Thermo Fisher Scientific, Inc.). The enzyme was removed by phenol-chloroform-isoamyl alcohol (PCIA) extraction followed by chloroform-isoamyl alcohol (CIA) extraction. The nature of the dsRNA was further confirmed by treatment with ShortCut RNase III (New England Biolabs, Inc.) (see Fig. S1 in the supplemental material). The dsRNA was extracted from 125 mg (fresh weight) of mycelia to load into a lane for gel electrophoresis. Electrophoretic separation of dsRNA was performed with a 1.0% (wt/vol) agarose gel in 0.5× Tris-borate-EDTA (TBE). For size estimation, purified genomic dsRNA of mycoreovirus 1/S10ss (66) was used.

Northern blot analysis was performed as follows. The electrophoresed dsRNA was capillary blotted onto a Hybond-N^+^ membrane (GE Healthcare, Ltd.) in 20× SSC (1× SSC is 0.15 M NaCl plus 0.015 M sodium citrate). After overnight blotting, dsRNA on the membrane was denatured as described previously by Sun et al. (67). Briefly, the membrane was immersed in a solution containing 50 mM sodium hydroxide (NaOH) and 10 mM NaCl for 5 min and then washed twice in 2× SSC. dsRNA on the membrane was cross-linked by UV irradiation and hybridized with digoxigenin (DIG)-11-dUTP-labeled cDNA probes. Hybridization and immunodetection of DIG were performed according to the manufacturer’s instructions (F. Hoffmann-La Roche, Ltd.). DIG-labeled probes were prepared by using PCR DIG labeling mix (F. Hoffmann-La Roche, Ltd.) with the primers listed in Table S3 at http://www.rib.okayama-u.ac.jp/pmi/Supplemental%20Material.html and plasmids containing cDNA for the HadV1 genome.

### Sequencing of HadV1 dsRNA.

To screen novel polymyco-related viruses, mixtures of dsRNA from three fungal isolates were subjected to NGS analysis. The three isolates included the F. oxysporum isolate 7n, the Alternaria alternata isolate 4a, and the *A. alternata* isolate A-16 (28). The methods and resultant total reads were described previously (28). NGS contigs showing homology to the genomic sequences of polymycoviruses were mined by local BLASTX using viral reference data sets provided by the National Center for Biotechnology Information (NCBI). The correspondence of the three polymyco-related segments to dsRNA from isolate 7n was confirmed by RT-PCR.

The complete genomic sequence of HadV1 was determined by Sanger sequencing of clones from a random cDNA library and by 3′ RLM-RACE. The random cDNA library was prepared by a PCR-based method (68) with dsRNA templates. 3′ RLM-RACE with dsRNA templates was performed according to a previously reported method (69) to determine the terminal sequences. The HadV1-specific primers used for RT-PCR in RLM-RACE are listed in Table S3 at the URL mentioned above. The consensus sequence of each terminus was determined with at least five RLM-RACE clones. Multiple-sequence alignment of the terminal nucleotide sequences was conducted using ClustalW2 (https://www.ebi.ac.uk/Tools/msa/clustalw2/). The plausible ORFs were predicted using Open Reading Frame Finder (https://www.ncbi.nlm.nih.gov/orffinder/) provided by the NCBI.

### Phylogenetic analysis.

The phylogenetic relationship between HadV1 and the known viruses was analyzed based on the deduced amino acid sequences of RdRP. The analysis involved RdRP amino acid sequences of 14 polymycoviruses (10 assigned and 4 unassigned), 13 members of the family *Caliciviridae*, 6 members of the family *Astroviridae*, and 12 members of the family *Partitiviridae*. The full names of the viruses and GenBank accession numbers of their RdRP sequences are shown in Table 1 and in Table S2 at http://www.rib.okayama-u.ac.jp/pmi/Supplemental%20Material.html, except for PjPmV1 (see above). Some amino acid sequences were trimmed before multiple-sequence alignment, according to the experimentally determined polyprotein cleavage site or putatively annotated RdRP domain. The sequences were aligned by using PSI-Coffee (T-COFFEE, ver. 11.00) (70). The obtained multiple-sequence alignment is shown in Text S1 (Clustal format) and Text S2 (FASTA format) at the URL mentioned above. Some of the results are shown in Fig. 2A and Fig. S4. The phylogenetic tree was constructed by using the maximum likelihood method with the best-fit model LG+G+I in MEGA X with default settings (71). The phylogeny was tested by the bootstrap method with 500 iterations. The complete-deletion option, which eliminates all positions containing gaps and missing data, was applied for tree construction. All genera in each family were covered and properly clustered (Fig. S5).

### Detection of HadV1 RNA by RT-PCR.

Conidia generated on mycelia of F. oxysporum isolate 7n were suspended in sterilized water and spread onto PDA medium to obtain single conidial subisolates. Whether 7n single conidial subisolates were HadV1 positive or negative was determined by mycelial direct one-step RT-PCR (Fig. S6B) as previously described (72), with slight modification. Briefly, mycelial contents were attached to the edge of a toothpick and rubbed on the inside bottom of a 0.2-ml PCR tube. The mycelial contents in the tube were mixed with 5 μl of PrimeScript one-step RT-PCR kit ver.2 (Dye Plus) (TaKaRa Bio, Inc.) containing primers specific for HadV1 RNA1 (RdRP-encoding segment), listed in Table S3 at the URL mentioned above. RT-PCR was performed with the following program: 50°C for 30 min; 94°C for 2 min; 28 cycles of 94°C for 30 s, 60°C for 30 s, and 72°C for 30 s; and 72°C for 2 min.

The presence of a full set of HadV1 genomic segments in conidial HadV1(+) subisolates was confirmed by RT-PCR (Fig. 3A). Total ssRNA was extracted from mycelia cultured on cellophane-PDA for 2 days as previously described (73). Total ssRNA (100 ng) was subjected to an RT reaction using Moloney murine leukemia virus reverse transcriptase (Thermo Fisher Scientific, Inc.) with random-hexamer primers, according to the manufacturer’s instructions, on a half-scale. PCR was performed using Quick *Taq* HS DyeMix (Toyobo Co., Ltd.) mixed with the respective specific primers (see Table S3 at the URL mentioned above) and 0.5 μl of the 10-fold-diluted cDNA products in a total reaction mixture volume of 10 μl. The PCR cycle conditions were according to the manufacturer’s standard 3-step program.

### Virus particle purification.

Crude virus particle fractions were obtained by a conventional method as previously described (Fig. 4A) (27, 28, 30–32). Fungal mycelia were cultured on cellophane-PDA for 5 to 7 days at room temperature. Scraped mycelia were ground in liquid nitrogen (LN_2_) using a mortar and pestle. The frozen mycelial powder was homogenized in 4 volumes (vol/wt) of 0.1 M sodium phosphate (pH 7.0) and 0.004 volumes (vol/wt) of 2-mercaptoethanol. The homogenate was mixed with a one-quarter volume of CCl_4_ and immediately centrifuged at 2,000 × *g* for 20 min at 4°C. This CCl_4_ extraction step was repeated once. The supernatant was mixed with 8% (wt/vol) PEG (*M*_w_, 6,000) and 1% (wt/vol) NaCl and stirred for 2 h on ice. The solution was centrifuged at 15,000 × *g* for 20 min at 4°C. The resultant pellets were suspended in 0.05 M phosphate buffer (pH 7.0). The suspension was again centrifuged at 8,000 × *g* for 10 min at 4°C to remove undissolved PEG. The supernatant was overlaid onto a 20% (wt/vol) sucrose cushion in a 5:2 volume ratio and ultracentrifuged at 100,000 × *g* for 1.5 h at 4°C with an Optima L-100K ultracentrifuge (Beckman Coulter, Inc.). After ultracentrifugation, the resultant pellet was dissolved in 0.05 M sodium phosphate (pH 7.0). The dsRNA contained in each fraction, equivalent to the content in 250 mg of mycelia, was purified by PCIA and CIA extraction followed by cellulose column chromatography.

For milder purification (Fig. 5A), the frozen mycelial powder was homogenized with 0.05 M sodium phosphate (pH 7.0) as mentioned above. The supernatant was filtered through Miracloth (Merck Group) and centrifuged at low speed (3,000 × *g*) for 20 min at 4°C to remove cell debris. The supernatant was optionally subjected to CCl_4_ extraction (Fig. 5C, bottom). The supernatant was ultracentrifuged at 100,000 × *g* for 1.5 h at 4°C with an Optima L-100K ultracentrifuge. The resultant pellet was suspended in 0.05 M sodium phosphate (pH 7.0). The dsRNA in each fraction was purified as mentioned above. The ssRNA in each fraction was enriched by PCIA and CIA extraction followed by 2 M lithium chloride (LiCl) precipitation.

### RNase A assay.

The experimental procedure for the RNase A assay is illustrated in Fig. 6A. Fungal mycelia were cultured on cellophane-PDA for 5 days at room temperature and powdered in the presence of N_2_. The mycelial powder was homogenized in 4 volumes (vol/wt) of 0.05 M sodium phosphate (pH 7.0) and filtered through Miracloth, as in the above-mentioned milder virus purification steps (Fig. 5A). The filtrate was treated with RNase A (10 μg/ml) (Sigma-Aldrich Co., LLC) for 30 min at 37°C. The solution was extracted with phenol to inactivate RNase A, followed by PCIA and CIA extraction. The supernatant was separately used for dsRNA extraction and RT-PCR. The dsRNA in 700 μl of the supernatant was isolated by cellulose column chromatography and subjected to gel electrophoresis. For RT-PCR, total ssRNA in 400 μl of the supernatant was precipitated in 2 M LiCl. The rinsed pellet was dissolved in 1 ml of pure water, and 1 μl of the ssRNA solution was subjected to a 10-μl RT-PCR mixture using PrimeScript one-step RT-PCR kit ver.2 (Dye Plus) (TaKaRa Bio, Inc.) and specific primers. The PCR was run by using the same program as the one mentioned above. Primers used for virus detection are listed in Table S3 at http://www.rib.okayama-u.ac.jp/pmi/Supplemental%20Material.html. F. oxysporum elongation factor 1 alpha (*ef1*α) mRNA (64) and *P. janthinellum* β-tubulin mRNA (*benA*) were employed as internal controls for RT-PCR, in which primer sets EF-1 and EF-2 (64) and Bt2a and Bt2b (74) were used, respectively (see Table S3 at the URL mentioned above).

### Data availability.

The complete nucleotide sequence of the HadV1 genome was deposited in the EMBL/GenBank/DDBJ database under accession numbers LC519840 to LC519850.
