# Supplementary figures and images for: Hadaka Virus 1: a Capsidless Eleven-Segmented Positive-Sense Single-Stranded RNA Virus from a Phytopathogenic Fungus, Fusarium oxysporum
Source: mBio. 2020 May 26;11(3):e00450-20. doi: 10.1128/mBio.00450-20 (PMC7251205; doi:10.1128/mBio.00450-20)

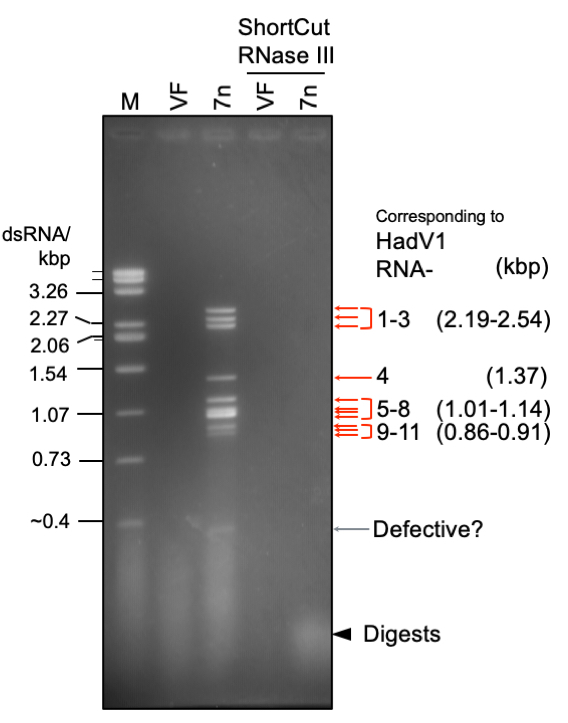

Supplement: FIG S1 [file mBio.00450-20-sf001.jpg]

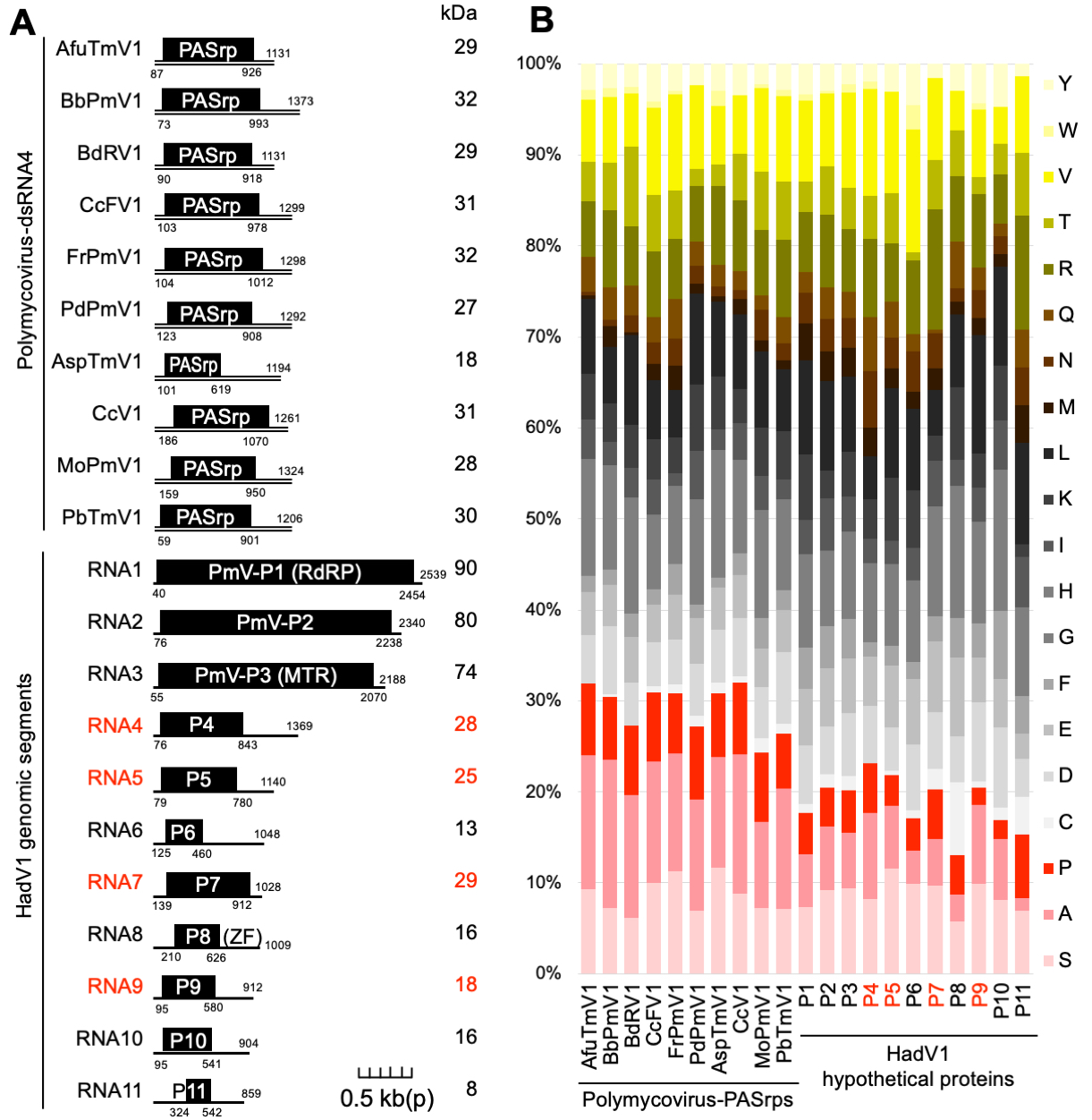

Supplement: FIG S2 [file mBio.00450-20-sf002.jpg]

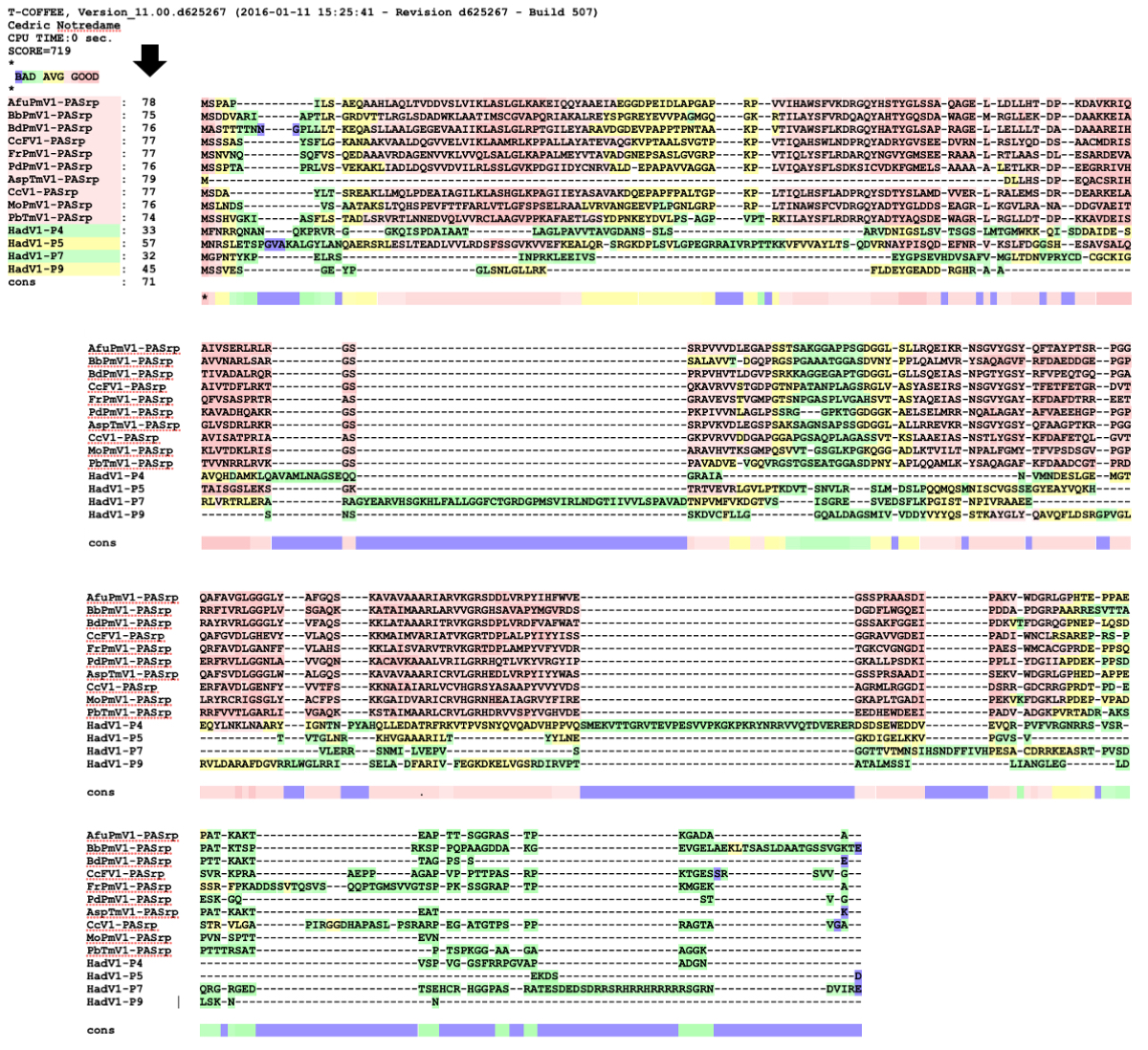

Supplement: FIG S3 [file mBio.00450-20-sf003.jpg]

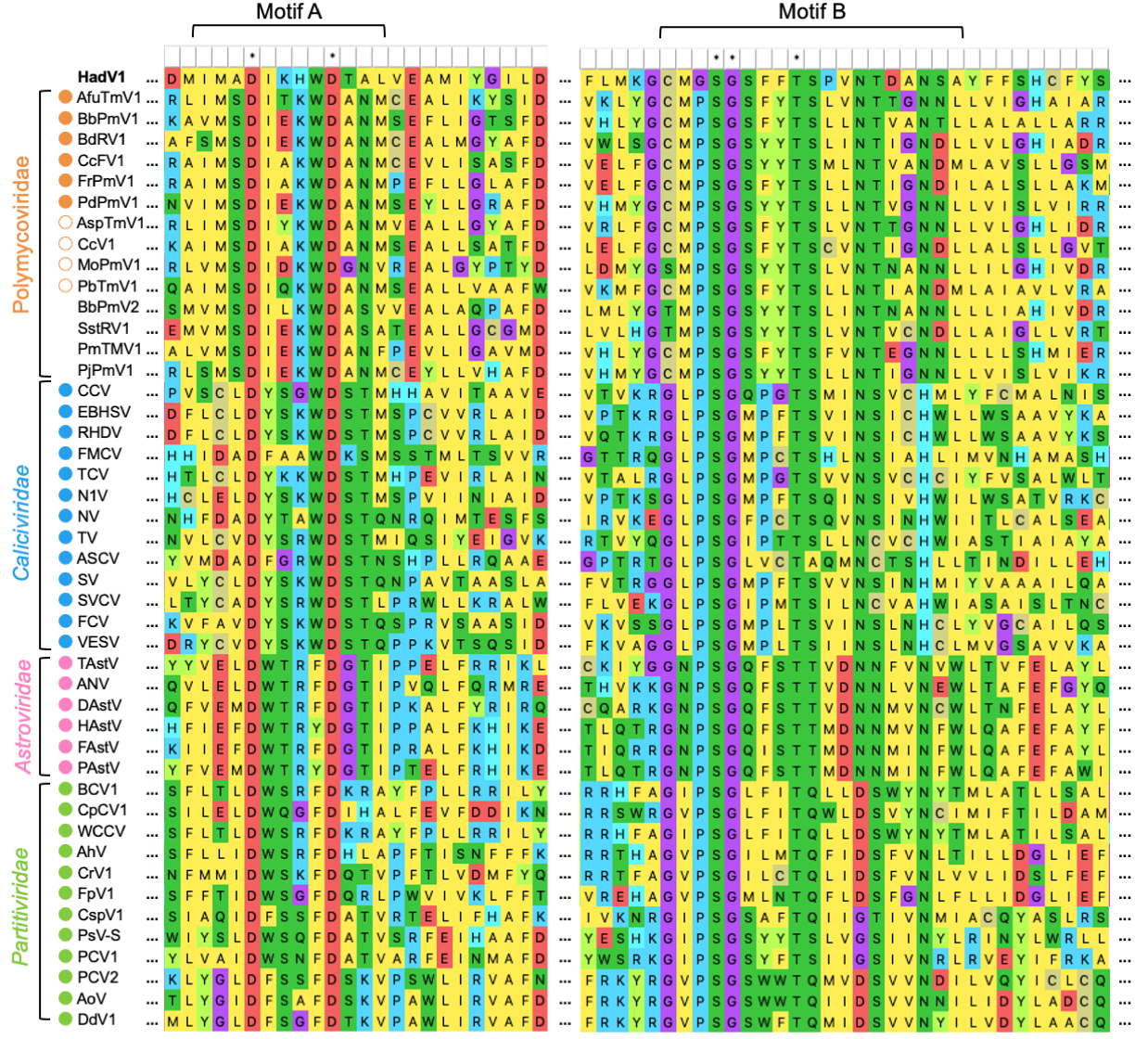

Supplement: FIG S4 [file mBio.00450-20-sf004.jpg]

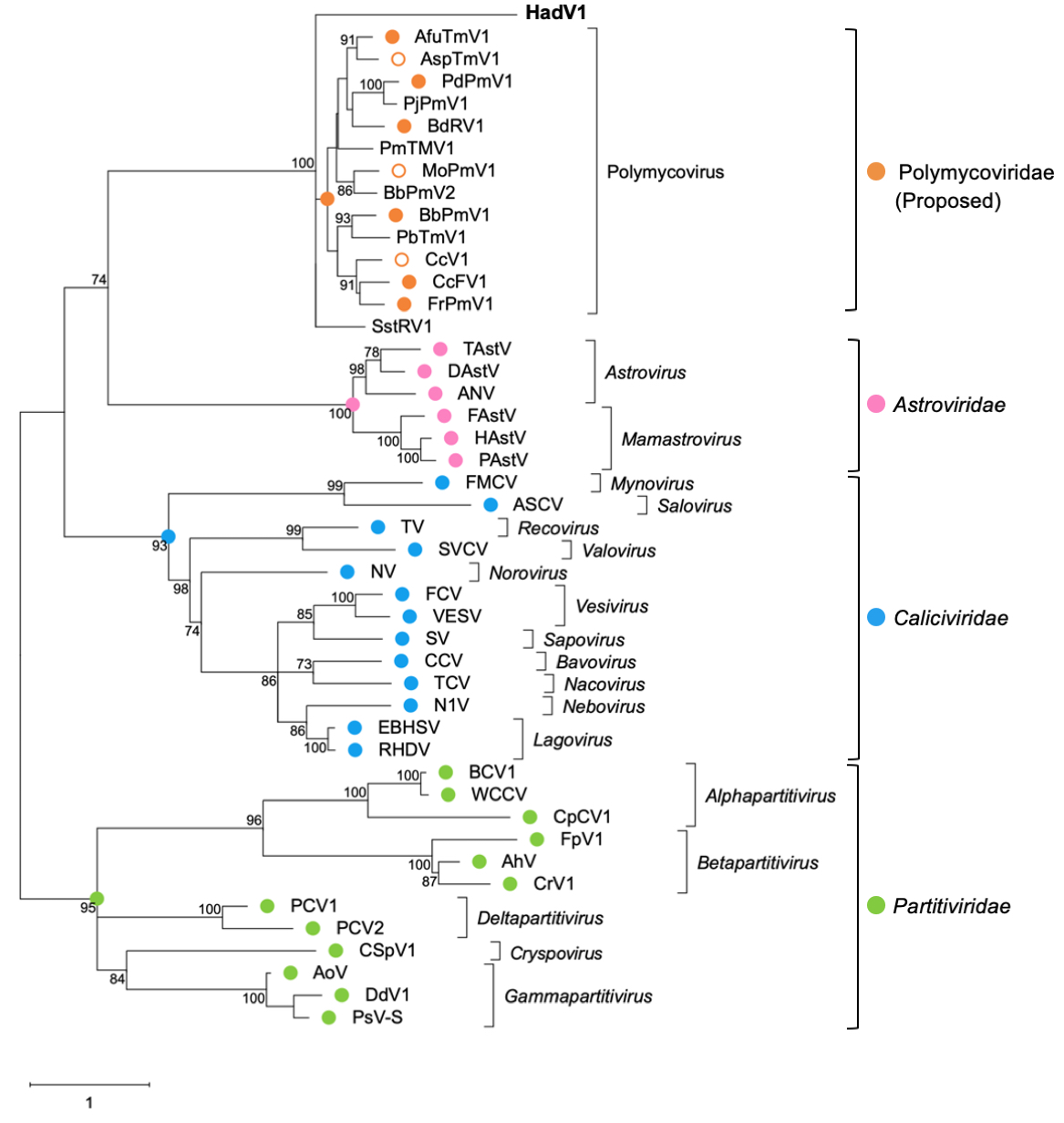

Supplement: FIG S5 [file mBio.00450-20-sf005.jpg]

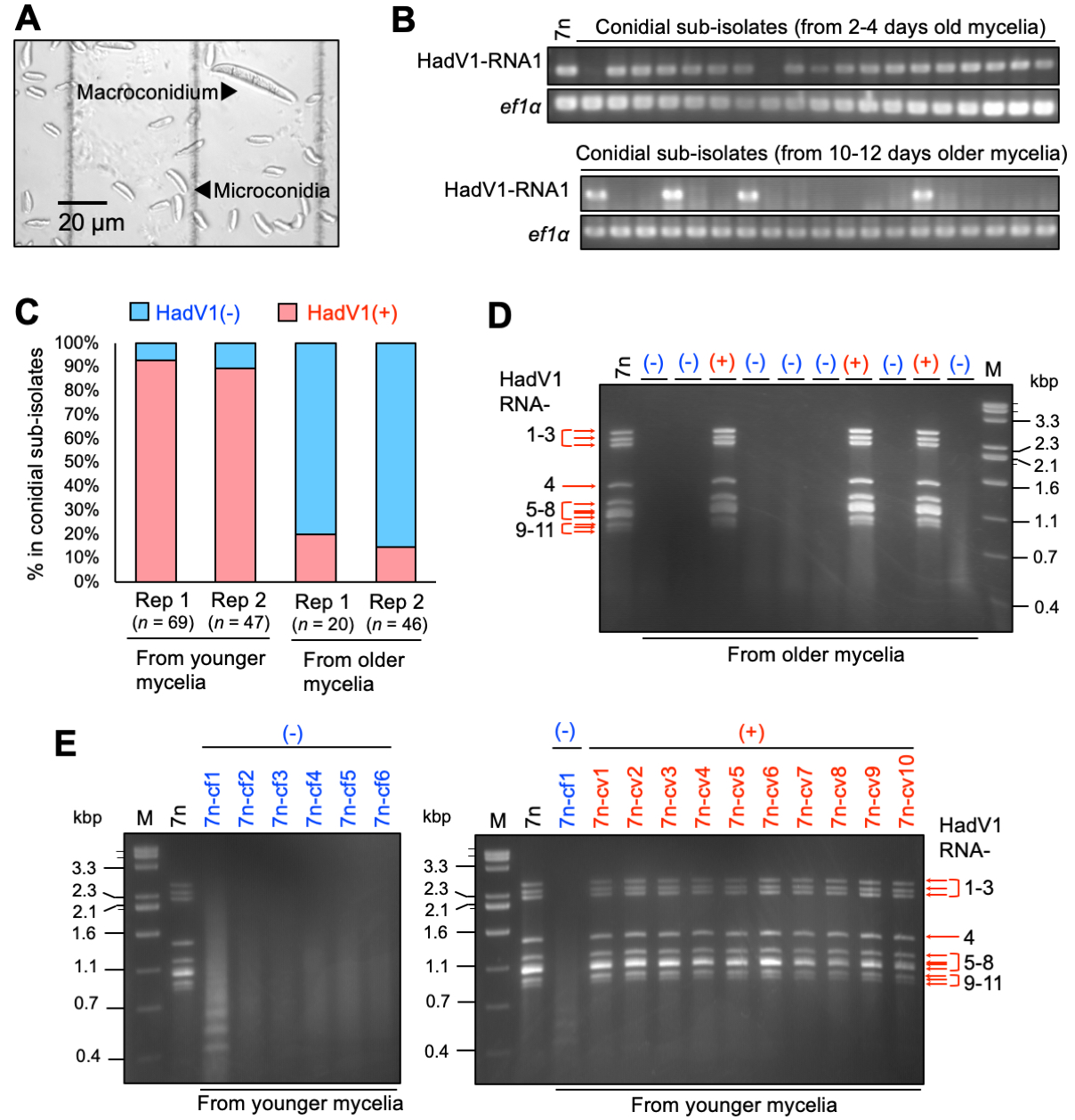

Supplement: FIG S6 [file mBio.00450-20-sf006.jpg]

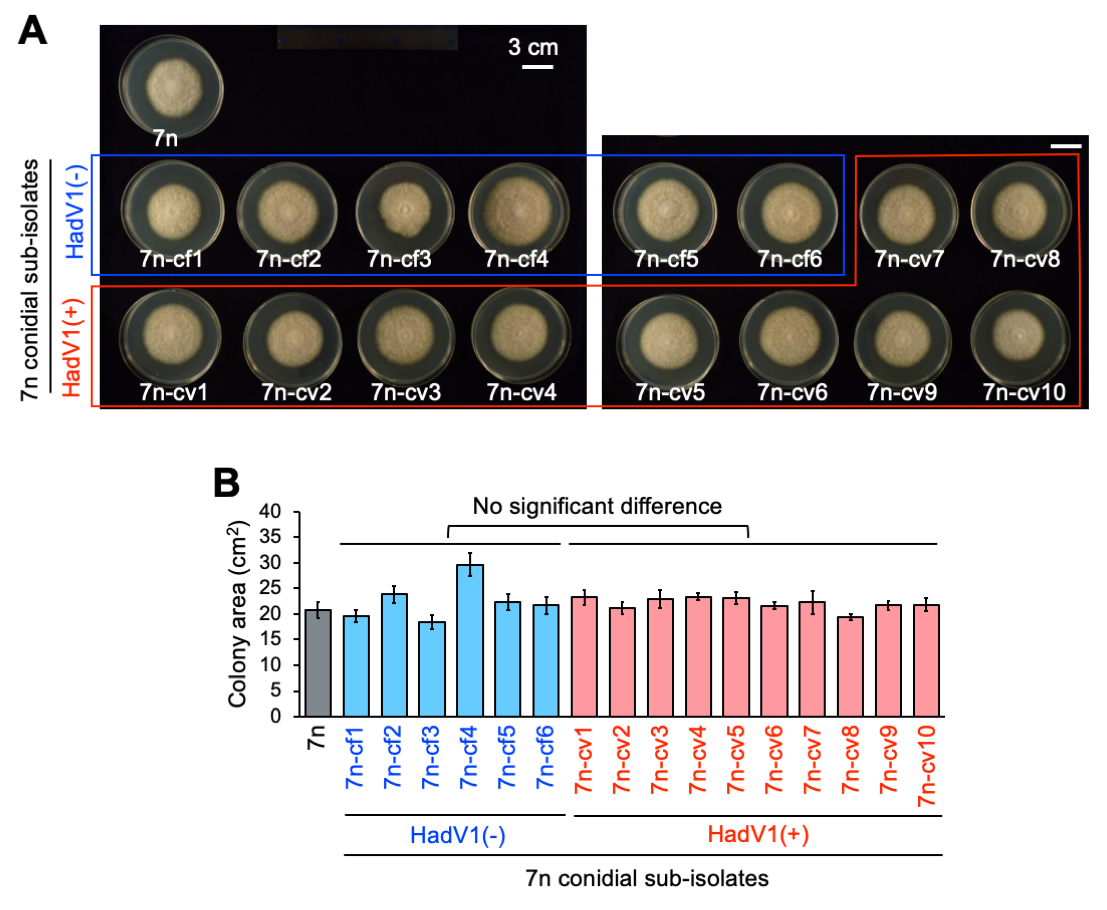

Supplement: FIG S7 [file mBio.00450-20-sf007.jpg]

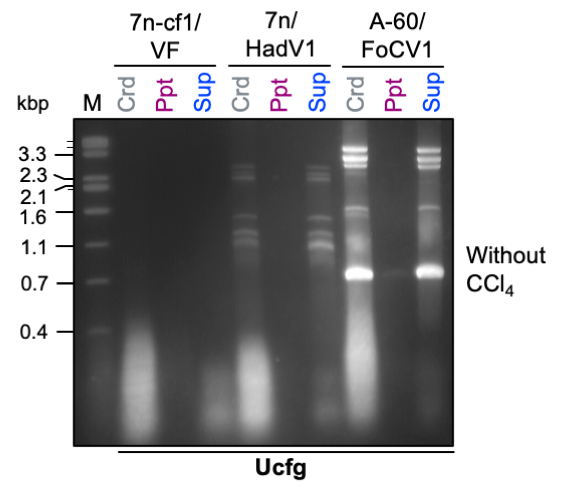

Supplement: FIG S8 [file mBio.00450-20-sf008.jpg]
